# A Case of Hypokalemia Caused by the Consumption of Licorice-Containing Herbal Tea

**DOI:** 10.7759/cureus.86154

**Published:** 2025-06-16

**Authors:** Promy Saha, Emmanuel Aydin-Ghormoz, Kelly Beers, Sidrah Abid

**Affiliations:** 1 Department of Medicine, Khulna Medical College Hospital, Khulna, BGD; 2 Department of Medicine, Albany Medical Center, New York, USA; 3 Department of Nephrology, Albany Medical Center, New York, USA

**Keywords:** diabetes mellitus type 2, hypokalemia, licorice, pseudohyperaldosteronism, sglt2 inhibitors

## Abstract

Hypokalemia can have many etiologies, of which licorice toxicity is notable but often overlooked. Licorice is being increasingly used as a component in herbal formulations and holistic remedies. We present a case of licorice-induced hypokalemia due to herbal tea formulation in a patient with a history of type 2 diabetes mellitus and metastatic adenocarcinoma. This case highlights a unique presentation of pseudo-hyperaldosteronism due to licorice consumption with concurrent use of a sodium-glucose cotransporter (SGLT2) inhibitor, requiring a high index of suspicion for diagnosis. Glycyrrhetinic acid (GA) is one of the active metabolites of licorice, which is responsible for intracellular cortisol accumulation and activation of mineralocorticoid receptors. Therefore, in addition to removing the offending agent, mineralocorticoid receptor antagonists can also be used in treatment.

## Introduction

Potassium distribution throughout the body is meticulously regulated by a network of hormones and cellular mechanisms. Hypokalemia can arise from inadequate intake but can also occur due to abnormal potassium losses for a variety of reasons, including exposure to offending agents such as licorice [1]. Licorice is an ancient herbal drug used in various food products such as candies, teas, and thirst quenchers [2]. Numerous studies have shown that active compounds in licorice exhibit antitumor, antimicrobial, antiviral, anti-inflammatory, and immunoregulatory properties; many of these findings are only experimental and limited to clinical studies [3]. For instance, liquiritigenin, a flavanone found in licorice, can act as a glucose-lowering agent in diabetics [4]. However, licorice overconsumption may cause more harm than benefit, as evidenced by its potential effects on potassium homeostasis.

Potassium is filtered by the glomerulus and mostly reabsorbed in the proximal tubule and ascending limb of Henle. Urinary potassium excretion is regulated by secretion in the aldosterone-sensitive distal nephron, primarily via apical potassium channels influenced by sodium reabsorption and activation of mineralocorticoid receptors under the influence of aldosterone [5]. When consumed in large quantities, glycyrrhetinic acid in licorice may cause overactivation of mineralocorticoid receptors [6], subsequently producing a state of pseudo-hyperaldosteronism, manifested by hypokalemia, metabolic alkalosis, and hypertension. Here, we present a case of hypokalemia caused by consumption of a licorice-containing herbal tea in a patient concurrently taking a sodium-glucose cotransport inhibitor (SGLT2i) for type 2 diabetes mellitus (T2DM).

## Case presentation

A woman in her 70s was referred to our institution for evaluation of hypokalemia. She had a history of type 2 diabetes mellitus and adenocarcinoma of the pyriform sinus with metastatic lymphadenopathy that had been treated with cisplatin-based chemotherapy (which was discontinued several months prior when she developed neuropathy). At the time of presentation, she was receiving radiation therapy. She had experienced progressive difficulty swallowing over the previous three months and had a prophylactic percutaneous endoscopic gastrostomy (PEG) tube placed six weeks prior to presentation, although she had not yet initiated use of this tube. One week prior to presentation, her oral intake had sharply declined due to the development of severe odynophagia, and she was only taking liquids and small amounts of scrambled eggs by mouth. Her liquid intake included an herbal tea recommended by a friend to help soothe her throat pain. She presented to her oncologist’s office for routine scheduled follow-up, where lab work showed a potassium level of 2.0 mEq/L (ref. 3.5-5.3 mEq/L), and she was referred to our hospital for further management.

In our emergency department, her potassium level was 2.2 mEq/L, and she was admitted to the internal medicine service. Laboratory analysis was also significant for a sodium level of 142 mEq/L (135-145 mEq/L), magnesium level of 2.0 mg/dL (1.5-3.5 mg/dL), phosphorus level of 2.1 mg/dL (2.5-4.5 mg/dL), serum bicarbonate 24 mEq/L (22-28 mEq/L), blood urea nitrogen (BUN) of 4 mg/dL (7-22 mg/dL), creatinine of 0.49 mg/dL (0.7-1.3 mg/dL), and anion gap of 21 mmol/L. Venous blood gas showed a pH of 7.46 (7.32-7.43). Blood pressure was 148/71. She was treated with a total of 320 mEq of potassium chloride by both intravenous and oral routes over the next 24 hours, with improvement of serum potassium level to 2.7 mEq/L. While her initial phosphorus level was mildly below the normal range, it increased to 2.6 mg/dL within eight hours, and it continued to be within the normal range for the rest of hospitalization. The nephrology service was consulted on hospital day 2 for assistance with management of refractory hypokalemia. Further history was obtained, and it was discovered that she was prescribed empagliflozin, an SGLT2i, for management of her T2DM and had continued taking this despite poor oral intake. She also admitted to drinking five to six cups of a commercially available herbal throat soothing tea daily for the prior six to eight weeks. This tea contained 820 mg of licorice root per tea bag.

Initially, plasma renin activity (PRA) was checked and was low at 0.167 ng/mL/hr (ref 0.167-5.380 ng/mL/hr). Aldosterone level was ordered but not sent. In the setting of SGLT2i use and poor intake, beta hydroxybutyrate level and anion gap were checked and both were elevated at 2.9 mmol/L and 21 mmol/L, respectively. Empagliflozin was held, and the patient received intravenous fluids. Two days later, beta hydroxybutyrate level and anion gap decreased to 1.5 mmol/L and 8 mmol/L, respectively. Concurrently, bicarbonate level rose to a peak of 39 mEq/L by hospital day 4, while the beta hydroxybutyrate level continued to fall. The bicarbonate level then normalized over the subsequent seven days. Potassium levels initially remained low despite aggressive PO and IV repletion, and spironolactone 25 mg daily was started on hospital day 4. This, in conjunction with ongoing potassium repletion, led to normalization of the patient’s potassium level by hospital day 10. Hospitalization was prolonged due to methicillin-sensitive *Staphylococcus aureus* (MSSA) bacteremia secondary to a chest wall port. She was discharged on hospital day 12 with prescriptions for spironolactone 25 mg daily and potassium chloride 40 mEq twice daily. Her potassium level was 3.6 mEq/L and stable. Empagliflozin was resumed upon discharge. She has been following with her oncologist and primary care provider, with no reported recurrence of these electrolyte and acid-base abnormalities.

## Discussion

**Figure 1:**
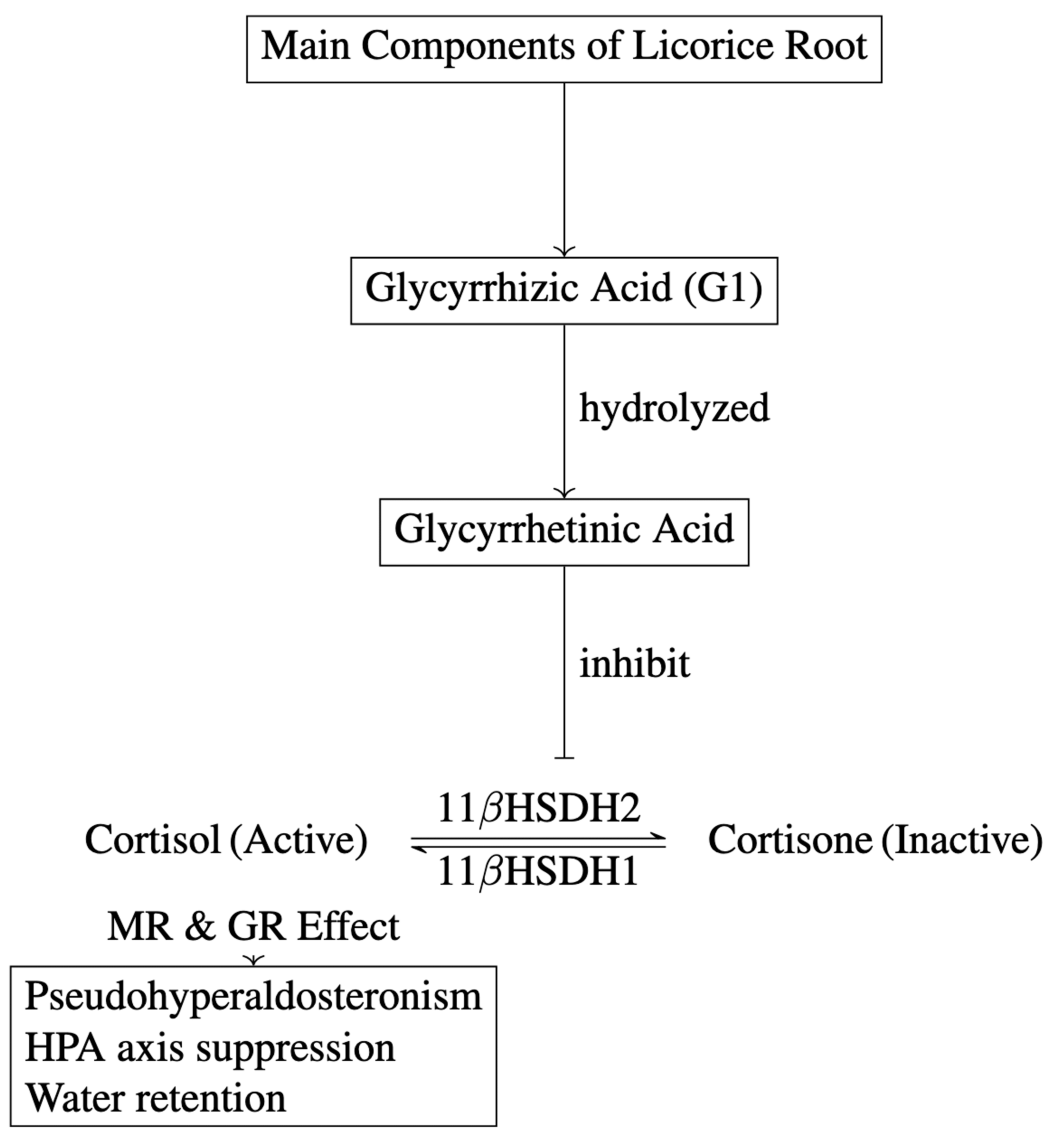
Metabolism of Licorice Root and Glycyrrhizic Acid (GI) Chart Image credit: Promy Saha. 11βHSDH1: 11-beta hydroxysteroid dehydrogenase type 1, 11βHSDH2: 11-beta hydroxysteroid dehydrogenase type 2, MR: mineralocorticoid receptor, GR: glucocorticoid receptor, HPA: hypothalamic-pituitary-adrenal axis.

The differential diagnoses for our patient revolved around etiologies of low-renin hypertension, such as Conn syndrome, or syndromes of apparent mineralocorticoid excess, including Liddle’s syndrome, deficiencies of enzymes such as 11-hydroxylase or 17-hydroxylase, and pseudo-hypoaldosteronism due to licorice toxicity [7]. In consideration of the patient’s low BUN and creatinine levels, which are indicative of poor oral intake or malnutrition, the possibility of nutritional deficiency contributing to her hypokalemia was carefully evaluated. While her initial phosphorus level was mildly low, it increased to 2.6 mg/dL within a short period and continued to be normal, including other electrolytes such as magnesium, except potassium. This trend suggests that although refeeding syndrome was a potential concern, it was not a significant contributor to the electrolyte abnormalities noted. The cause of severe, persistent hypokalemia and metabolic alkalosis with low renin and (presumed) aldosterone levels in our patient was due to excessive licorice consumption. Glycyrrhetinic acid, a metabolite in licorice, inhibits 11-beta hydroxysteroid dehydrogenase type 2, an enzyme that converts cortisol to cortisone in principal cells of collecting duct. This inhibition causes cortisol to accumulate intracellularly, activating mineralocorticoid receptors. Activation of mineralocorticoid receptors subsequently activates epithelial sodium channels (ENaC) on the luminal side of principal cells. Sodium flows intracellularly down its concentration gradient, leaving a total net negative charge behind in the tubular lumen. This charge drives potassium secretion into the urine via renal outer medullary potassium (ROMK) channels. Cortisol has a greater affinity for the mineralocorticoid receptor than aldosterone, and the elevated cortisol levels in the principal cells causes a “hypermineralocorticoid state” consisting of hypertension, hypokalemia, and metabolic alkalosis (Figure 1). As levels of renin and aldosterone are classically low in this state, this condition has been termed the syndrome of apparent mineralocorticoid excess (SAME). Both Liddle syndrome and congenital adrenal hyperplasia may also cause a SAME [8].

In the realm of ancient herbal medicines, licorice is one of the most widely used medicinal plants [9]. Our patient was consuming licorice dry root in an herbal tea as a holistic remedy for cancer-related symptoms. It contained about 820 mg of licorice root per tea bag, and she consumed five teabags per day on average. Glycyrrhetinic acid (GA), the hydrolytic metabolite of licorice, is the key active component responsible for inducing pseudo-hyperaldosteronism [10] which inhibits 11-hydroxysteroid dehydrogenase type 2 (11HSDH2), leading to increased levels of cortisol. On average, around 31.5 mg of GA can be found in a regular 250 mL cup of licorice tea [11].

While metabolic alkalosis always co-presents with hypokalemia in pseudo-hyperaldosteronism, it is noteworthy that the initial presentation of hypokalemia without metabolic alkalosis in this patient could be attributed to SGLT2i-induced euglycemic diabetic ketoacidosis with positive serum ketones (euglycemic diabetic ketoacidosis (Eu DKA)) on admission [12]. Moreover, SGLT2i may cause mild hypotension due to osmotic diuresis, particularly among elderly patients, with an incidence of 1.2%-1.5% [13], which helps explain the absence of elevated blood pressure in this licorice toxicity-mediated pseudo-hyperaldosteronism. To establish diagnosis, urinary GA can be measured, but it is not well studied [6]. In the absence of a gold standard diagnostic test, we rely solely on clinical presentation and detailed history to establish diagnosis of licorice toxicity.

It is worth mentioning that in this patient, history of throat adenocarcinoma and chemotherapy-induced anorexia could also potentially exacerbate hypokalemia [14] and increase susceptibility to licorice toxicity [15]. While licorice toxicity is a globally encountered phenomenon [16], there is currently no established treatment protocol. Potassium replacement therapy remains crucial as a primary intervention in addition to the removal of the offending agent [6]. Close monitoring is needed until the effects of licorice or other toxic agents wear off [17]. Mineralocorticoid receptor antagonist (MRA) spironolactone is a potential treatment option [18]. MRAs would block the mineralocorticoid receptor, in effect inactivating ENaC channel, thus counteracting the effects of licorice.

## Conclusions

The serum potassium level is tightly controlled and regulated through a series of hormones and mechanisms in the kidneys. Hypokalemia can be seen in pseudo-hyperaldosteronism that can be triggered by licorice. Herbal products often used for holistic medicine contain licorice and can cause toxicity. In this case report, we presented a case of an elderly female with a past medical history of type 2 diabetes mellitus and throat adenocarcinoma receiving chemotherapy, who presented with severe hypokalemia due to licorice consumption and concurrent SGLT2i use. While pseudo-hyperaldosteronism is typical of licorice toxicity, this patient presented with hypokalemia without alkalosis due to concurrent use of an SGLT-2 inhibitor, resulting in ketosis masking metabolic alkalosis. Clinicians should keep a high index of suspicion for licorice-induced pseudo-hyperaldosteronism, even in the absence of classical features of this condition.
